# Neutrophils and Neutrophil Extracellular Traps Drive Necroinflammation in COVID-19

**DOI:** 10.3390/cells9061383

**Published:** 2020-06-02

**Authors:** Bhawna Tomar, Hans-Joachim Anders, Jyaysi Desai, Shrikant R. Mulay

**Affiliations:** 1 Division of Pharmacology, CSIR-Central Drug Research Institute, Lucknow 226031, India; bhawnatomar415@gmail.com; 2 Division of Nephrology, Department of Medicine IV University Hospital LMU, 80336 Munich, Germany; Hans-Joachim.Anders@med.uni-muenchen.de; 3 Department of Rheumatology, Leiden University Medical Center, 2333 ZA Leiden, The Netherlands; jyaysidesai@gmail.com

**Keywords:** SARS-CoV-2, coronavirus, neutrophils, NETs, complement, thrombosis, MERS-CoV, necroinflammation

## Abstract

The COVID-19 pandemic is progressing worldwide with an alarming death toll. There is an urgent need for novel therapeutic strategies to combat potentially fatal complications. Distinctive clinical features of severe COVID-19 include acute respiratory distress syndrome, neutrophilia, and cytokine storm, along with severe inflammatory response syndrome or sepsis. Here, we propose the putative role of enhanced neutrophil infiltration and the release of neutrophil extracellular traps, complement activation and vascular thrombosis during necroinflammation in COVID-19. Furthermore, we discuss how neutrophilic inflammation contributes to the higher mortality of COVID-19 in patients with underlying co-morbidities such as diabetes and cardiovascular diseases. This perspective highlights neutrophils as a putative target for the immunopathologic complications of severely ill COVID-19 patients. Development of the novel therapeutic strategies targeting neutrophils may help reduce the overall disease fatality rate of COVID-19.

## 1. Introduction

The novel severe acute respiratory syndrome coronavirus (SARS-CoV)-2 was first discovered in Wuhan, China, and believed to have transmitted from bats to humans [1]. The SARS-CoV-2 has higher human-to-human transmission capabilities compared to the SARS-CoV and Middle East respiratory syndrome coronavirus (MERS-CoV) and has resulted in a pandemic. The World Health Organization has named the disease COVID-19: coronavirus disease-2019; since it was first reported in December 2019. Although SARS-CoV-2 affects lungs at first, it can extend to many organs, including the heart, kidneys, gut, blood vessels, and the brain [2]. 

The SARS-CoV-2 is closely related to the SARS-CoV since they have 80% similarity in genome sequence and seven conserved non-structural domains identified by protein sequence analysis [3,4]. Moreover, they both have a similar receptor-binding domain, and therefore both use the same cell entry receptor, i.e. angiotensin-converting enzyme II (ACE2) [5]. Subsequently, viral replication in combination with the subsequent antiviral immune response both contribute to the severity of COVID-19, which in some patients involves cytokine storm followed by severe inflammatory response syndrome (SIRS), sepsis, multi-organ failure, and death [6]. However, little is known about the immune pathomechanisms that trigger the cytokine storm during COVID-19. We propose that as part of the first line of the innate immune defense, neutrophils are critical for the exacerbation of the immune response, and that neutrophil extracellular traps (NETs)-related necroinflammation plays a central role in the development of the cytokine storm, sepsis and multi-organ failure during COVID-19.

## 2. ACE2 and Neutrophils

ACE2, a homolog of ACE and central negative regulator of the renin-angiotensin system is a type 1 integral membrane glycoprotein monocarboxypeptidase that converts angiotensin-II (AngII) to Ang-(1–7) and is constitutively expressed by the epithelial cells of the lungs, kidney, heart, and intestines on the outer surface [5,7]. Ang-(1–7) is a vasodilator that mediates anti-inflammatory, anti-proliferative, and anti-fibrotic effects through the Mas receptor [8]. Using ACE2-mutant mice, Imai, et al. demonstrated protective functions of ACE2 in acute respiratory distress syndrome (ARDS) [7]. They observed that ACE2 negatively regulates AngII, and thus, increases vascular permeability, lung edema, and the infiltration of neutrophils, partially mediated by the angiotensin 1 receptor (AT1R) [7]. Interestingly, SARS-CoV-infected mice or mice receiving injections of SARS-CoV spike protein showed an aggravated phenotype compared to ACE2-mutant mice, suggesting the contribution of ACE2 beyond being a mere receptor for SARS-CoV [9]. Similar to SARS-CoV, upon binding to ACE2, SARS-CoV-2 enters cells along with ACE2 leading to reduced ACE2 expression on the cell surface [5]. Therefore, the loss of ACE2 might contribute to the severity of ARDS during COVID-19 by increasing AngII- and AT1R-mediated vascular permeability, lung edema, and neutrophils infiltration [10].

How does ACE2 regulate the infiltration of neutrophils mechanistically? Sodhi et al. demonstrated that attenuation of pulmonary ACE2 activity leads to activation of des-Arg9 bradykinin (DABK)/bradykinin receptor B1 (BKB1R) axis, the release of pro-inflammatory chemokines e.g., C-X-C motif chemokine ligand 5 (CXCL5), macrophage inflammatory protein-2 (MIP2), CXCL1, and tumor necrosis factor (TNF)-α from airway epithelia, increased neutrophil infiltration, and exaggerated endotoxin-induced lung inflammation and injury [11]. The dynamic variation of pulmonary ACE2 was found essential to control neutrophilic inflammation, i.e., a balanced reduction of ACE2 while encountering a bacterial lung infection to recruit inflammatory neutrophils to combat the infection and later its recovery to restrict neutrophil accumulation to alleviate the inflammation by limiting interleukin (IL)-17 signaling by reducing STAT3 pathway activity [12]. Thus, ACE2 prevents the infiltration of neutrophils at the injury or infection site.

## 3. SARS-CoV-2, Neutrophils, and Necroinflammation in COVID-19

An increased neutrophil-to-lymphocyte ratio predicts severe illness in the early stage of SARS-CoV-2 infection, whereas neutrophilia frequently develops in COVID-19 patients in intensive care units [6,13,14,15,16]. Being part of the first line of innate immune defense, neutrophils have been thought to have protective roles during bacterial or fungal infections, where they kill bacteria or fungi by phagocytosis as well as NET formation [16]. However, their role in viral infections remains unclear. In murine SARS-CoV infection, neutrophils were dispensable for antibody-mediated clearance of SARS-CoV from pulmonary cells as well as the survival of SARS-CoV-infected mice [17,18]. On the other hand, continuous infiltration of neutrophils at the site of infection and their degranulation and release of NETs in response to microbial stimuli to raise an immune response produces exaggerated cytokines and chemokine that might result in the “cytokine storm” and contribute to the ARDS, SIRS and sepsis development during COVID-19 [6,14,19]. Higher levels of interleukin (IL)-1β, interferon-γ, CXCL10, monocyte chemoattractant protein-1, granulocyte colony-stimulating factor, monocyte inhibitory protein-1, and TNF-α were observed in COVID-19 patients requiring ICU admission [6,14]. A lung autopsy from a patient who succumbed to COVID-19 revealed an extensive neutrophil infiltration in pulmonary capillaries with extravasation into the alveolar space displaying acute capillaritis, as well as neutrophilic mucositis of the trachea indicating inflammation to the entire airway [20]. Moreover, SARS-CoV-2 infection of endothelial cells and the accumulation of inflammatory cells induced endothelitis in multiple organs, which may contribute to the systemic impaired microcirculatory function during COVID-19 [21] and to the phenomenon of the “happy hypoxia” [22]. 

**Figure 1:**
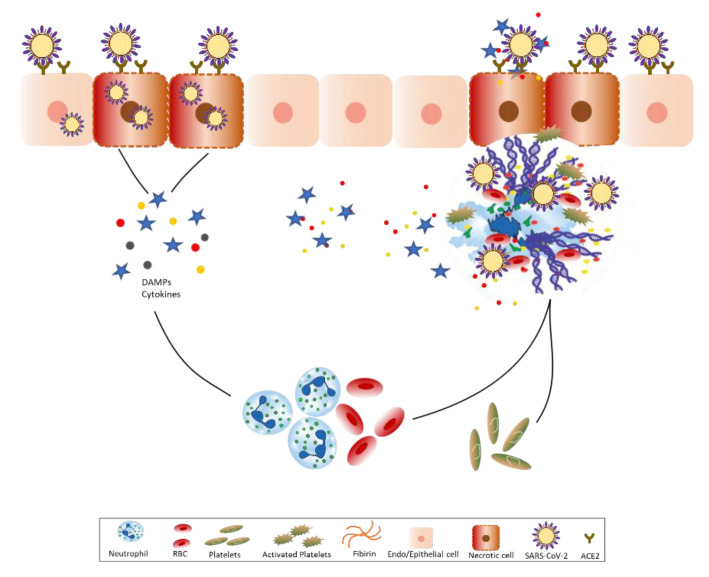
Neutrophils and neutrophil extracellular traps drive necroinflammation in COVID-19. The severe acute respiratory syndrome coronavirus-2 (SARS-CoV-2) binds to ACE2 and enter epithelial as well as endothelial cells along with it leading to reduced ACE2 expression that stimulates neutrophil recruitment. Subsequently, neutrophils undergo degranulation and NET formation releasing intracellular danger-associated molecular patterns, e.g., DNA, histones, neutrophil elastase that activate the pattern recognition receptors on surrounding immune and non-immune cells to induce cytokine secretion. The extracellular DNA released by NETs activates platelets and aggregated NETs provide a scaffold for binding of erythrocytes and activated platelets that promote thrombus formation. The extracellular histones present on NETs induce necrosis in epithelial or endothelial cells leading to the release of associated molecular patterns. This sets up an auto-amplification loop of necroinflammation that aggravate the disease severity during COVID-19. SARS-CoV-2 = severe acute respiratory syndrome coronavirus 2, ACE2 = angiotensin-converting enzyme 2, NET = neutrophil extracellular traps, DAMPs = danger-associated molecular patterns.

The SARS-CoV accessory protein open reading frames SARS3a induced multimodal necrotic cell death in epithelial cells [23]. Interestingly, SARS3a is conserved in SARS-CoV-2 [4], suggesting the engagement of similar pathomechanisms during COVID-19. Cellular necrosis as well as NET formation results in the release of several intracellular danger-associated molecular patterns that activate the pattern recognition receptors on the surrounding immune and non-immune cells resulting in more production of inflammatory cytokines and chemokines [24]. The release of NETs disperses histones, DNA, and granule proteins, such as myeloperoxidase, neutrophil elastase, cathepsin G, and proteinase 3, which results in severe tissue destruction, setting up the auto-amplification loop of necroinflammation [24,25] (Figure 1).

NETing neutrophils tend to form larger aggregates called “AggNETs” that drive the formation of thrombi in blood vessels [26]. Interestingly, high incidences of venous thrombosis are reported in COVID-19 [27]. The extracellular DNA released by NETs activates the platelets, and the AggNETs provide a scaffold for binding of the erythrocytes and activated platelets, which further promote the NET formation and set up a vicious cycle propagating thrombus formation [26]. NETs also activate the complement system. Myeloperoxidase, cathepsin G, and proteinase 3 activate properdin, factor B, and C3, three components of the alternative pathway required to induce the complement cascade [28]. Activated neutrophils also express properdin, factor B, and C3, suggesting an important role of neutrophils in complement activation. Of note, activation of the complement system has been reported in the severe COVID-19 patients [27]. Together, neutrophils infiltration and NETs formation drive necroinflammation during coronavirus infections (Table 1).

## 4. Diabetes, SARS-CoV-2, and Neutrophils

Many prevalent co-morbidities increase the severity and mortality of COVID-19 [14,27,39,40,41]. One of the most distinctive co-morbidities is diabetes mellitus [6]. Out of 1099 cases reported by Guan et al., 16.2% of patients with severe disease had a higher prevalence of diabetes compared to 5.7% of patients with the non-severe disease [14]. Case fatality was higher in COVID patients with diabetes [42]. This may be attributed to the dysfunctional innate immunity, as well as the exaggerated pro-inflammatory cytokine response in patients with diabetes [43]. Furthermore, higher glucose levels glycosylate and shed ACE2 [44] may contribute to the severity of ARDS during COVID-19 by increasing vascular permeability, edema, and neutrophils infiltration in DM patients. On the other hand, it was believed that patients with diabetes treated with ACE inhibitors and angiotensin-receptor blockers may develop increased ACE2 expression, which could further facilitate the cell entry of SARS-CoV-2 and aggravate the infection [40]. However, a recent study reported no association with the likelihood of COVID-19 positive test or severity of COVID-19 with renin-angiotensin system inhibitors [45]. Hyperglycemia in diabetes primes neutrophils to release NETs that might further contribute to the cytokine storm, SIRS, and sepsis in COVID-19 [43]. Besides, sugar-activated neutrophils produce S100 Calcium-binding proteins A8/A9 (S100A8/A9) that increased the production of thrombopoietin in the liver and subsequent thrombocytosis [46], which might contribute to thrombus formation in COVID-19. Th17-associated cytokine production promoted disease-predictive inflammation in DM [47]. Interestingly, a higher number of CCR6+ TH17 cells were found in the peripheral blood of COVID-19 patients, suggesting critical involvement of TH17 response [48]. Together, neutrophil-mediated cytokine storm leads to sepsis and subsequent multi-organ failure to aggravate the severity of COVID-19 disease.

## 5. Cardiovascular Diseases, SARS-CoV-2, and Neutrophils

Cardiovascular diseases, including coronary heart disease, cardiomyopathy, arrhythmias, myocardial injury, and hypertension are other distinctive co-morbidities of COVID-19 that have higher overall mortality rates [14,42,49]. Especially, the extent of myocardial injury correlated with cardiac dysfunction, arrhythmias, and fatal outcome of COVID-19 [49]. ACE2 exerts vasodilatory effects through Ang-(1–7) and the Mas receptor [8]. Therefore downregulation of ACE2 upon SARS-CoV-2 cell entry induces vasoconstriction and subsequent hypertension. Subsequent ACE2-mediated neutrophil infiltration, as well as NET formation, might be responsible for the exaggerated inflammatory response, which in turn contributes to the development of cardiovascular diseases, e.g., thrombosis, atherosclerosis, and endothelial injury, etc. One in five hospitalized COVID-19 patients showed increased troponin, brain natriuretic peptide, lymphopenia, and inflammation markers, such as c-reactive protein, IL-1β, and IL-6 in the early course of the disease suggesting cardiac injury [49,50]. Recently, NET-related endothelial cell injury was reported to contribute to vascular pathology in pulmonary hypertension [39]. Moreover, IL-1β promoted the thrombus formation via NET-associated tissue factor during atheroembolic events during cardiovascular diseases [51,52]. Furthermore, increased neutrophil elastase activity was reported to contribute to obesity, insulin resistance, and related inflammation [53]. Interestingly, the presence of obesity in metabolic associated fatty liver disease increased the severity of COVID-19 six-fold [41]. All these reports indicate the involvement of neutrophils and related necroinflammation in the pathology and severity of COVID-19.

## 6. Summary and Perspectives

To summarize, neutrophils play a central role in the immunopathology of COVID-19. SARS-CoV-2 infection, as well as downregulation of ACE2 upon the cell entry of SARS-CoV-2 triggers neutrophil infiltration in the lungs. Necrotic cell death of alveolar epithelial cells, as well as NET formation, releases damage-associated molecular patterns and alarmins in the surrounding extracellular space, which induce production of pro-inflammatory cytokines and vice versa, setting up a loop of necroinflammation that is responsible for the cytokine storm and sepsis. NETting neutrophils cause endothelial injury and necroinflammation via complement activation, as well as promote the venous thrombus formation during COVID-19. Underlying co-morbidities in COVID-19 patients, e.g., diabetes and cardiovascular diseases enhance the neutrophilic inflammation and thereby severity of COVID-19. Therefore, the development of novel therapeutic strategies targeted at neutrophils, e.g., inhibitors of neutrophil recruitment or NET formation may help reduce the overall disease mortality rate of COVID-19.

## Figures and Tables

**Table 1:** Evidence for neutrophil-mediated necroinflammation in coronavirus infections.

Virus	Evidence for Involvement of Neutrophils	Reference
SARS-CoV-2	High levels of markers of NETs, e.g., cell free-DNA, myeloperoxidase-DNA, and citrullinated histone 3 in sera from severely ill patients	[19]
	High neutrophil-to-lymphocyte ratio cause ARDS in patients	[13,15,29]
	Neutrophil infiltration in pulmonary capillaries with extravasation into the alveolar space	[20]
	High neutrophil-to-lymphocyte ratio and D-dimer levels in patients	[30]
SARS-CoV	C3 mediated neutrophil recruitment during disease progression in mice	[31]
	Neutrophils infiltration in lungs during the late phase of infection in mice	[32]
	Neutrophils count correlate with the cytokine storm in patients	[33]
	Higher levels of neutrophil chemokine IL-8 found in patients	[34]
	Neutrophilia is associated with the severity of disease in patients	[35]
MERS-CoV	Neutrophil-mediated innate inflammatory response in human DPP4 knock-in mice	[36]
	Increased neutrophils contribute to leukocytosis, an indicator of disease severity and fatality in patients	[37]
	Increased release of ROS caused extensive pulmonary lesions and increased the disease severity in marmosets	[38]

SARS-CoV = severe acute respiratory syndrome coronavirus, MERS-CoV = Middle East respiratory syndrome coronavirus, NET = neutrophil extracellular trap, ARDS = acute respiratory distress syndrome, C3 = complement factor 3, ROS = reactive oxygen species.
